# The seed morphospace, a new contribution towards the multidimensional study of angiosperm sexual reproductive biology

**DOI:** 10.1093/aob/mcae099

**Published:** 2024-06-22

**Authors:** Angelino Carta, Filip Vandelook, Santiago Ramírez-Barahona, Si-Chong Chen, John Dickie, Tina Steinbrecher, Costas A Thanos, Angela T Moles, Gerhard Leubner-Metzger, Efisio Mattana

**Affiliations:** Department of Biology, Botany Unit, University of Pisa, Pisa, Italy; Meise Botanic Garden, Meise, Belgium; Department of Botany, Universidad Nacional Autónoma de México, Mexico City, México; State Key Laboratory of Plant Diversity and Specialty Crops, Wuhan Botanical Garden, Chinese Academy of Sciences, Wuhan, China; Royal Botanic Gardens, Kew, Wakehurst, Ardingly, West Sussex, UK; Royal Botanic Gardens, Kew, Wakehurst, Ardingly, West Sussex, UK; Department of Biological Sciences, Royal Holloway University of London, Egham, UK; Section of Botany, National and Kapodistrian University of Athens, Athens, Greece; Evolution & Ecology Research Centre, School of Biological, Earth and Environmental Sciences, UNSW Sydney, Sydney, NSW 2052, Australia; Department of Biological Sciences, Royal Holloway University of London, Egham, UK; Royal Botanic Gardens, Kew, Wakehurst, Ardingly, West Sussex, UK

**Keywords:** Angiosperms, comparative seed biology, seed coat, embryo, endosperm, gymnosperms, macroevolution, seed mass

## Abstract

**Background:**

The evolutionary success of flowering plants is associated with the vast diversity of their reproductive structures. Despite recent progress in understanding angiosperm-wide trends in floral structure and evolution, a synthetic view of the diversity in seed form and function across angiosperms is lacking.

**Scope:**

Here we present a roadmap to synthesize the diversity of seed forms in extant angiosperms, relying on the morphospace concept, i.e. a mathematical representation which relates multiple traits and describes the realized morphologies. We provide recommendations on how to broaden the range of measurable traits beyond mass, by using key morphological traits representative of the embryo, endosperm and seed coat but also fruit attributes (e.g. dehiscence, fleshiness). These key traits were used to construct and analyse a morphospace to detect evolutionary trends and gain insight into how morphological traits relate to seed functions. Finally, we outline challenges and future research directions, combining the morphospace with macroevolutionary comparative methods to underline the drivers that gave rise to the diversity of observed seed forms.

**Conclusions:**

We conclude that this multidimensional approach has the potential, although still untapped, to improve our understanding of covariation among reproductive traits, and further elucidate angiosperm reproductive biology as a whole.

## INTRODUCTION

**Fig. 1.**
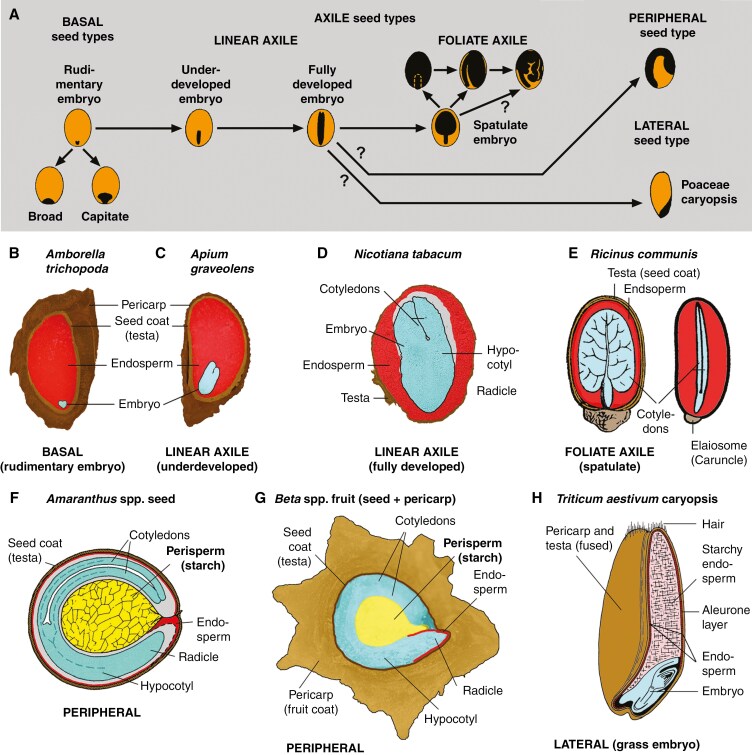
Morphological diversity of seeds and fruits. (A) Seed type evolution and internal morphology. Simplified scheme of seed and embryo types, and working model of their evolutionary relatedness in angiosperms (Martin, 1946; Linkies *et al.*, 2010; Baskin and Baskin, 2014). Examples for angiosperm seed and fruit types: (B) basal seed with rudimentary embryo (light blue) embedded in abundant endosperm tissue (red) (Fogliani *et al.*, 2017). Embryo (2*n*, maternal:paternal = 1:1) and endosperm (3*n*, maternal:paternal = 2:1) are products of double fertilization, seed coat (2*n*) is maternal tissue of the ovule (Linkies *et al.*, 2010). (C) Linear axile type with underdeveloped (small) embryo embedded in abundant endosperm tissue (Walker *et al.*, 2021). (D) Linear axile type with fully developed embryo embedded in abundant endosperm tissue (Finch-Savage and Leubner-Metzger, 2006). (E) Foliate axile type with thick spatulate cotyledons. Elaiosome as external morphological modification of the testa to aid seed dispersal by ants [J. Sachs (1887), *Vorlesungen über Pflanzen-Physiologie*, Verlag Wilhelm Engelmann, Leipzig]. (F) Peripheral seed type in which the embryo is curved around the central perisperm (2*n*, maternal tissue of the nucellus, yellow); example for seed as diaspore (Baskin and Baskin, 2019; Nakabayashi and Leubner-Metzger, 2021). (G) Example for fruit as diaspore with peripheral seed type encased by a thick pericarp (2*n*, fruit coat, maternal tissue of the flower). (H) Typical cereal caryopsis, with pericarp and testa fused, lateral seed type [K. Esau (1953), *Plant Anatomy*, Wiley & Sons, New York].

Several innovations in sexual reproduction involving an extensive diversification of reproductive structures, particularly in their dispersal units (Stebbins, 1951; Takhtajan, 1991), contributed to the success of angiosperms in terrestrial ecosystems (Benton *et al.*, 2022). This variation is well documented by the ontology and morphology of angiosperms’ dispersal units, or diaspores, which are represented by true seeds, one-seeded fruits such as achenes and cypselas, whole fruits, or part of fruits such as woody endocarps (Fig. 1). With the exception of fleshy and dehiscent fruits, these structures are commonly – and thus also hereafter in this study – referred to as ‘seeds’.

Substantial progress in resolving angiosperm phylogenetic relationships (APG, 2016; Soltis *et al.*, 2018) and the availability of extensive trait databases have provided a robust framework for tracing plant strategies (Díaz *et al.*, 2016; Vojtko *et al.*, 2022) and flower evolution (Chartier *et al.*, 2014; Sauquet *et al.*, 2017; López-Martínez *et al.*, 2023). These studies have greatly enhanced understanding of the correlations and trade-offs that have helped to shape the evolution of plant form and function. However, macroevolutionary studies focusing on seed and fruit morphology are scarce and tend to analyse individual traits separately. The most commonly analysed trait is seed mass, a multifunctional trait that influences many aspects of species’ regeneration strategy (Table 1), including the number of seeds produced per fruit/plant per year, dispersal syndrome and early seedling survival (Moles *et al.*, 2005; Sims, 2013; Carta *et al.*, 2022*b*). Other studies have focused on embryo size, a key trait related to germination timing in the environment (Forbis *et al.*, 2002; Vandelook *et al.*, 2012), and the seed coat, which plays an important role in seed dormancy, protection and persistence in the soil (Chen *et al.*, 2020; Pausas and Lamont, 2022). Here, we introduce the seed morphospace, a multivariate approach to synthesize the diversity of seed form and functions, advancing our understanding of angiosperm reproductive biology.

**Table 1.** Key morphological traits used to construct the seed morphospace, their functional role and relevant data sources/references.

Seed component	Trait	Type of variable (units/categories)	Definition	Functional significance	Source
Embryo/endosperm	Embryo size ratio	Quantitative (adimensional)	Embryo length to seed length ratio	Germination timing (higher embryo to seed ratio is associated with shorter germination timing)	F. Vandelook *et al.* (in prep.)
Embryo	Embryo type	Discrete (axile, basal, peripheral)	Position and structure of the embryo	Germination timing and modality of storage	Martin (1946), Baskin and Baskin (2014)
Endosperm	Perisperm	Binary	Storage tissue of maternal origin (2*n*)	Alternative storage tissue	Watson and Dallwitz (1992), https://www.delta-intkey.com/angio/index.htm http://www.mobot.org/MOBOT/research/APweb/
Seed coat	Seed coat ratio	Quantitative (adimensional)	Seed coat mass divided by whole seed mass	Fire tolerance, persistence in soil, resistance to pathogens and predators (higher value corresponds to higher resistance)	Chen *et al.* (2020)
	Fruit type	Discrete (dehiscence, indehiscent [dry] or fleshy)	Presence of extra-seed covering layers	Persistence/dispersal	Watson and Dallwitz (1992), https://www.delta-intkey.com/angio/index.htm
	Seed mass	Quantitative (mg)	Dry mass of an average dispersal unit	Dispersal distance; seed persistence; light detection; early seedling establishment; predation likelihood, number of seeds produced per unit canopy per year.	https://ser-sid.org/; Moles (2018)

A morphospace is a type of configuration space, usually visualized by means of multivariate ordinations, in which organisms are placed at points within the space according to their set of traits (Budd, 2021). The statistical description of forms through morphospaces, widely used in zoology (Raup, 1966; Foote, 1994; see also literature cited in Chartier *et al.*, 2014 and Budd, 2021), allows the assessment of realized morphologies among theoretical possibilities and to evaluate how phenotypic diversity (disparity) is distributed among lineages, by measuring the spread of taxa within the morphospace. A multidimensional approach can provide insights into the extent of correlation among traits and whether a given trait can limit or channel evolvability in others (Beldade *et al.*, 2002). Morphospaces represent an effective method to measure plant morphological diversity in a multivariate framework (Oyston *et al.*, 2016; Clark *et al.*, 2023), as shown for other reproductive features such as flowers (Chartier *et al.*, 2014) and pollen (Jardine *et al.*, 2022).

Seeds have multiple key functions in angiosperm reproduction, including dispersal, persistence and germination (Grubb, 1977; Primack, 1987; Finch-Savage and Leubner-Metzger, 2006; Baskin and Baskin, 2014). Seed morphological traits, together with the biophysical and biochemical properties of seeds, determine their specific ecological function (Steinbrecher and Leubner-Metzger, 2018). Thus, the application of a morphospace approach could help to link key seed functions to the corresponding morphological traits. Previous studies of seed morphospaces have proven their utility, but these focused on one or a few lineages at the subfamily and order levels (see Lu *et al.*, 2010; Benedict *et al.*, 2016). However, as we discuss in the next sections, when trying to construct a seed morphospace for the entire diversity of angiosperms, challenges arise mainly due to the different ontology of the dispersal units (e.g. true seed or fruit; Fig. 1), to the vast variety of their external structures (e.g. appendages or wings; Fig. 1), and their physiological development before and after dispersal. Thus, an angiosperm-wide morphospace requires handling and integrating the huge morphological variation observed among species (and even greater among families; Nikolaeva, 2004) in their internal and external seed morphology (Martin, 1946; Harper *et al.*, 1970; Fig. 1).

In this viewpoint, we present a roadmap to describe and synthesize the diversity of seed forms across extant angiosperms and illustrate the need for a seed-based conceptual and operational framework, starting from the morphospace approach. We first (1) review the diversity of seed morphological traits and provide recommendations on how to maximize trait coverage across lineages by focusing on a few key morphological traits. Then, as a proof-of-concept, we (2) construct and analyse the first seed morphospace at family level, describing and relating the seed forms across the angiosperm tree of life. By analysing the results of the constructed morphospace, we then (3) show how this approach is a powerful tool to detect possible patterns of morphological trends across lineages and prove how morphological traits can be related to specific seed functions through the morphospace. Finally, to disentangle the relationships between seed morphology and the underlying evolutionary processes that ultimately give rise to observed forms and functions, we (4) outline challenges and future research directions, to combine the seed morphospace with macroevolutionary comparative approaches, to provide a new contribution toward an integrated view of the angiosperm reproductive biology.

## RECOMMENDATIONS FOR MEASURING SEED FORMS BEYOND SEED MASS

Seeds (Fig. 1) have been frequently characterized in terms of their size (Sims, 2013) or mass (Moles *et al.*, 2005), a trait that has been linked to several reproductive functions (Table 1). However, contrasting evidence has been reported about the ecological significance of this trait (see Larson and Funk, 2016; Moles 2018, and literature cited therein), suggesting that it is unlikely to capture solely the full variation in seed functional morphology within and among species. Thus, efforts to study the diversity of seed forms must extend beyond seed mass and incorporate a multivariate set of traits for elucidating functions and interactions among seed morphology, plant organs, interacting organisms and the environment.

The seeds of angiosperms are usually composed of three interconnected structurally and genetically different components (Linkies *et al.*, 2010; Fig. 1):

(1) the embryo (2*n*), representing the sporophyte of the next generation;
(2) the endosperm (3*n*) or the perisperm (2*n*), acting as storage tissues;
(3) the seed coat (2*n*) and, in indehiscent fruits, the pericarp (2*n*, fruit layers), both exclusively of maternal origin, serving as protective layers upon seed dispersal (Takhtajan, 1991; Eriksson *et al.*, 2000; Huss and Gierlinger, 2021).

These three primary components can be viewed as distinct developmental and functional modules but, given their strong integration (Linkies *et al.*, 2010), selective pressures are expected to simultaneously act upon all three modules (Stebbins, 1951; Floyd and Friedman, 2000). We present here four recommendations, to guide the sampling strategy to maximize the statistical integration of traits and enhance the identification of major trends in seed trait (co)variation across lineages.

Key morphological traits for the seed morphospace should include embryo, endosperm or perisperm, and seed coat and fruit layers. A non-exhaustive list of seed coat (and fruit layers) traits includes surface structure, shape, colour and appendages (Martin, 1946; Harper *et al.*, 1970; Takhtajan, 1991). However, such external characteristics often vary markedly among species or genera from the same family, which may lead to difficulties in scoring such traits due to the absence of a type of trait. A way to overcome this issue, which could be generalizable to the whole of angiosperms, is to measure the seed coat mass (Wu *et al.*, 2019) and the dispersal structure investment, calculated as the mass of the dispersal structure per seed or the proportion of the mass of the dispersal structure relative to the total diaspore mass (Thomson *et al.*, 2018). While external characteristics of seeds are extremely variable, their internal morphology is less commonly investigated (Visscher *et al.*, 2022) although can be usually categorized based on relative embryo size, embryo position and structure within the seed (Martin, 1946; Linkies *et al.*, 2010; Vandelook *et al.*, 2012; Baskin and Baskin, 2014).

Fruit attributes should be included in the set of traits being sampled. This is necessary because seeds are dispersed either on their own or encapsulated by fruit layers (Bobrov and Romanov, 2019; Huss and Gierlinger, 2021). Examples for traits of dispersed dry fruits to be measured include the thickness of the pericarp (Fig. 1B, C, G), or the presence/absence of appendages that aid dispersal such as wings or persistent bracts. The nature of the dispersal unit can vary even within a specific genus or family (Fig. 1F, G) or even within individuals of the same species (heteromorphism) as in *Aethionema arabicum* (L.) Andrz. ex DC. (Brassicaceae, Arshad *et al.*, 2019), which produces dehiscent fruits releasing true seeds by dehiscence, and single-seeded indehiscent fruits released by abscission in the same plant. To add a further layer of complexity, some fruit/seed traits are typical of a single family such as in the Poaceae caryopses where pericarp and seed coat (testa) are fused (Fig. 1H). Further to this, in many Poaceae diaspores the caryopsis is in addition encased by various hulls (e.g. in *Avena fatua* L.; Holloway *et al.*, 2021). To account for this complexity in morphological features, we suggest to score independently for the presence or absence of each trait state, for example by scoring fruit type (dehiscence, fleshiness) (see ‘Construction and visualization of the seed morphospace’).

Understanding major trends of seed diversity and evolution requires a comparative, phylogenetic framework applicable to a broad range of species (Garamszegi, 2014). The inclusion of species in such a framework (iii) should be designed based on trait and molecular data sampling (Sauquet *et al.*, 2017; Ramírez-Barahona *et al.*, 2020; López-Martínez *et al.*, 2023).

Finally, bearing in mind the huge variation in seed forms observed across species, it is unlikely that any integrative study will be able to capture the full range of variation of seeds. Many morphological traits may be found in only a few lineages or species within a specific lineage (Corner, 1976; Barthlott, 1981; Takhtajan, 1985; Werker, 1997). While these features are of high utility in taxonomic classification, accounting for all of them to construct the morphospace is challenging. This is why we propose to (iv) focus on a few key morphological traits with a defined functional role (Table 1) and representative of the three structurally and genetically different components described above.

## THE USE OF THE SEED MORPHOSPACE IN EVOLUTIONARY ECOLOGY

**Fig. 2.**
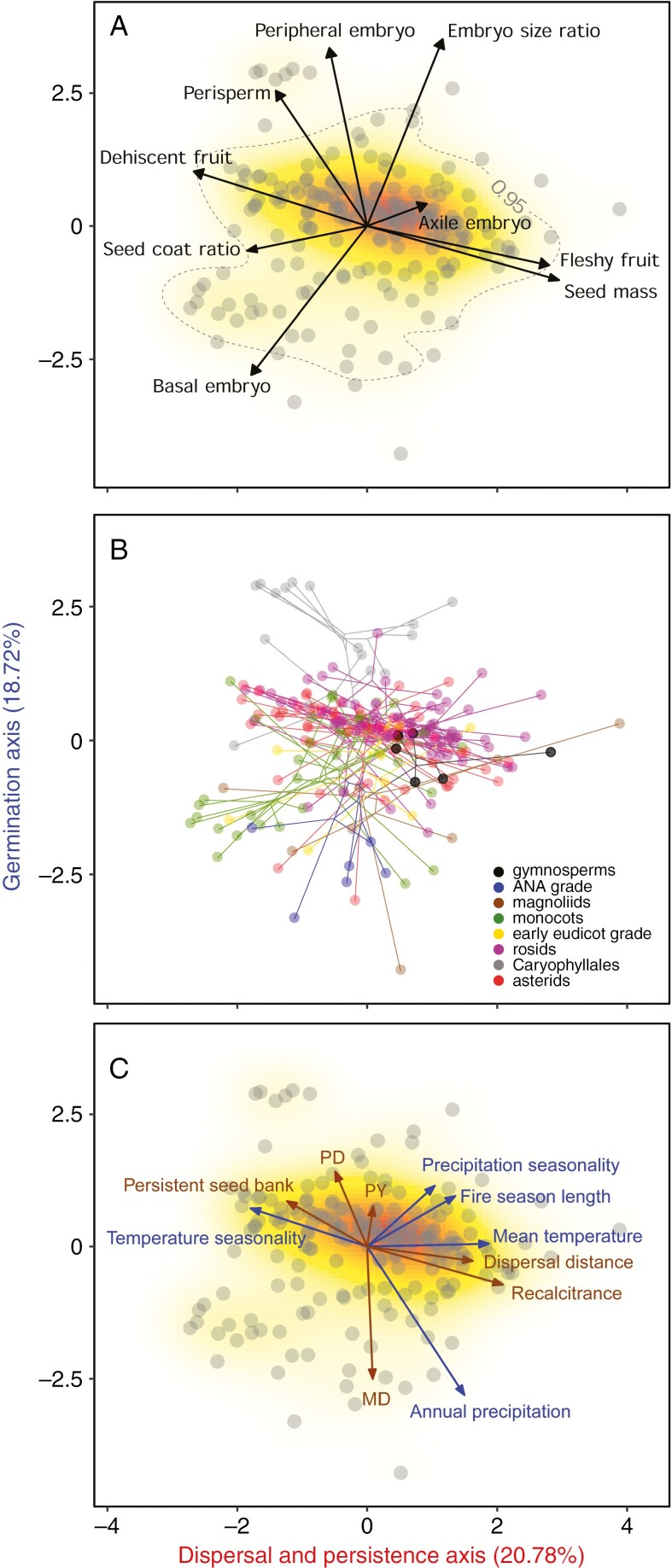
The seed morphospace. (A) Projection of 203 angiosperm families (dots) onto the plane defined by the first two dimensions of the seed morphospace. The first dimension is interpreted as a ‘dispersal and persistence axis’ while the second dimension is interpreted as a ‘germination axis’. Solid arrows indicate direction and weighing of vectors representing the key morphological traits [two continuous: embryo size ratio, seed coat ratio, and three discrete traits: embryo type (Axile, Basal, Peripheral), perisperm presence, fruit type (dry dehiscent versus dry indehiscent, fleshy) plus the seed mass as a fundamental morphological trait integrating multiple functions; see Table 1]. The colour gradient indicates regions of highest (red) to lowest (white) occurrence probability of species in the trait space, with the contour line indicating the 0.95 quantile (kernel density estimation). Gymnosperms are also considered for comparative purposes. (B) Seed morphospace extension 1: the seed phylomorphospace showing the location of main lineages in the morphospace. Projection of the phylogenetic tree into the 2D plane to illustrate the magnitude and the direction of morphological evolution within and among major APG groups. Adaptive peaks or stochastic processes influence lineages to evolve within restricted areas, with concomitant increases in crossing of phylogenetic branches in the plot. (C) Seed morphospace extension 2: the seed functional spectrum in relation to seed functional traits [mean dispersal distance, soil seed bank persistence, recalcitrance probability and dormancy classes (MD, morphological dormancy; PD, physiological dormancy; PY, physical dormancy)] and climatic variables included as supplementary variables to gain insight into how morphological traits map in relation to key seed functions (dispersal, persistence, germination); see Table 2.

We present here the first seed morphospace for flowering plants, representative of the whole phylogenetic diversity of angiosperms, using multivariate ordination of traits recorded at the family level (Fig. 2A). In addition, we also account for phylogenetic relatedness among families to illustrate the magnitude and direction of lineage morphological evolution within the seed morphospace (Fig. 2B). Finally, we examine whether and how these morphological traits relate to key seed functions (Fig. 2C). However, we would like to stress that this first approach to construct a seed morphospace serves only as a working example to visualize major seed-trait (co)variation among angiosperm families and not for hypothesis testing.

**Table 2.** Seed functional traits and climatic variables considered as supplementary variables (all projected onto the seed morphospace), with related sources/references.

Variable	Type of variable (units/categories)	Functional significance	Source
Mean seed dispersal distance	Quantitative (m)	Spatial dispersal, moving seeds away from parental plants	Chen *et al.* (2020)
Recalcitrance probability	Quantitative (adimensional)	Desiccation tolerance	Wyse and Dickie (2017)
Soil seed bank persistence	Discrete (persistent/transient)	Persistent seeds remain viable in the soil for at least a year or beyond the start of the second germination season	Gioria *et al.* (2020)
MD, morphological dormancy (germination prevented by a small or underdeveloped embryo at the time of dispersal)	Discrete (dormant/non-dormant)	Maximization of successful plant recruitment in sites subjected to seasonal or interannual variation in habitat suitability for seedling establishment	Baskin and Baskin (2014), Rosbakh *et al.* (2023)
PD, physiological dormancy (germination prevented by the seed’s internal balance of ABA/GA)	Discrete (dormant/non-dormant)	Maximization of successful plant recruitment in sites subjected to seasonal or interannual variation in habitat suitability for seedling establishment	Baskin and Baskin (2014), Rosbakh *et al.* (2023)
PY, physical dormancy [germination prevented by seed (fruit) coat unpermeability]	Discrete (permeable/impermeable)	Maximization of successful plant recruitment in sites subjected to seasonal or interannual variation in habitat suitability for seedling establishment; fire tolerance; defence against predators	Baskin and Baskin (2014), Paulsen *et al.* (2014), Rosbakh *et al.* (2023)
Climatic variables (BIO1, BIO4, BIO12, BIO15)	Quantitative	Ecosystem energy (BIO1, BIO12) and its seasonality (BIO4, BIO15)	https://chelsa-climate.org/
Potential fire season length (FSL)	Quantitative (months)	Local wildland fire activity	Senande-Rivera *et al.* (2022)

ABA/GA, abscisic acid/gibberellin.

### Construction and visualization of the seed morphospace

We used data for key morphological traits listed in Table 1 for 203 angiosperm families (48 % of extant angiosperm families globally), which represent all major APG (2016) groups (ANA-grade, magnoliids, monocots, rosids, asterids; Supplementary Data Table S1). We collated data on traits related to the three seed components (embryo, endosperm, seed coat/fruit layers): embryo size ratio (embryo length to seed length ratio), seed coat ratio (seed coat mass divided by whole seed mass), embryo type and perisperm presence (Table 1). To further account for the diversity in dispersal units (e.g. true seeds versus fruits), we included fruit type information (dehiscence, fleshiness). We also included seed mass as a fundamental trait integrating multiple functions in angiosperms’ life-history (Moles *et al.*, 2005). Quantitative traits available at the species level were averaged for each family. For discrete traits, we annotated the presence of character states for each family while for multistate categorical traits, we scored independently for the presence or absence of each state of such traits to account for intra-familiar polymorphism. This was the case for fruit traits, as species with either dehiscent or indehiscent and dry or fleshy fruits can be present within a family (see Table S1). All traits were fully scored across all sampled taxa, with the exception of seed coat ratio, for which we gathered data for 86 families (45 % of the total included in the matrix). Moreover, as angiosperms are not the only group of plants bearing seeds, we compared angiosperm traits with those of extant gymnosperms by gathering data for six gymnosperm families (50 % of extant families). Data were analysed using multiple factor analysis with the function mfa from the R package FactoMineR (Lê *et al.*, 2008), which allows the analysis of both continuous and discrete variables.

The multivariate ordination enabled the visualization of the position of each family within the seed morphospace (Fig. 2). Ideally, the seed morphospace should be built at the species level, but such high-quality data are currently not readily available from accessible databases. Despite this limitation, 39 % of trait variation was captured in the two-dimensional family-level morphospace here presented, highlighting major angiosperm-wide trends of realized seed morphologies based on trait (co)variation (Fig. 2A). The first axis of the morphospace reflects a trade-off between the size of the whole seed and the protective layer(s), whereas the second axis reflects the relative length of the embryo at dispersal and its position and structure within the seed. Although much seed morphological variation remains unexplained (determined partly by the choice to operate at the family level), the observed seed morphologies are strongly concentrated within the ordination space, indicating coordination and trade-offs among traits, convergent evolution or developmental restriction to seed evolution.

### Seed morphospace extension 1: the phylomorphospace

The most distinct property of any morphospace is how different species or lineages occupy (are distributed across) this space (Budd, 2021). To illustrate possible patterns of morphological evolution across angiosperm lineages and build a phylomorphospace (Revell, 2012), we projected an updated family-level, time-calibrated phylogenetic tree (Ramírez-Barahona *et al.*, 2020) onto the morphospace (Fig. 2B). Phylogenetic signal in morphospace occupancy was quantified using the function physignal from the R package geomorph (Baken *et al.*, 2021); this quantifies whether phylogenetically closely related taxa are more closely located in the morphospace than expected by chance.

The occupancy of the seed morphospace does not appear to be random. Families belonging to the same major lineage cluster together (Fig. 2B), with a phylogenetic signal (*K* = 0.86) indicating similar morphospace occupancy among more closely related families (Supplementary Data Fig. S1). For example, monocot families occupy the bottom part of the morphospace whereas the ANA-grade, although also occupying the bottom part, appears to be more restricted. Families in the Caryophyllales are spread across the upper part of the morphospace and segregated from other angiosperm groups, which is due to their seeds being usually perispermic and with a large, peripheral embryo. Overall, these patterns suggest that phylogenetic relatedness and some degree of trait conservatism (Losos, 2008) are probably responsible for the shared morphologies observed among closely related families. At the same time, the densely occupied regions of the seed morphospace (the overlap of groups in the space) also highlight that similar trait combinations have evolved independently in multiple lineages (Sims, 2013), or conserved due to developmental restrictions. In particular, rosid and asterid families largely overlap along the central part of the morphospace, which can be attributed to the comparatively similar embryo size ratio and variance in seed mass and fruit types. Gymnosperms are not clearly separated from angiosperms, suggesting a comparatively similar seed structure between both spermatophyte clades, despite their separation in terms of seed mass (Moles *et al.*, 2005).

To quantify morphological diversity across angiosperms, we measured disparity among taxa within the major APG groups with two different indexes measuring the spread of the occupied areas in morphospace (mean pairwise distances and range). Angiosperm groups exhibit differences in morphological disparity (Supplementary Data Fig. S2). According to both disparity indices, the ANA-grade, early eudicots, rosids and asterids have the lowest levels of disparity, whereas magnoliids, monocots and Caryophyllales have the highest. It is interesting to note that this pattern also reflects the levels of flower disparity observed across angiosperm groups (Chartier *et al.*, 2014), with the main exception concerning the ANA-grade that instead shows the highest flower morphological disparity.

Altogether, the seed morphospace highlights that, even when limited to a few key morphological traits scored at the family level (Table 1; Fig. 2), this type of multivariate approach can detect main trends and constraints acting on seed evolution. Nevertheless, accounting for the phylogenetic relatedness is not sufficient to evaluate the degree of morphological innovation versus the tendency to retain ancestral traits (Sansalone *et al.*, 2022). As we discuss below, comparative approaches using explicit evolutionary models at the species level are needed to disentangle the relative contribution of these two alternative processes (Garamszegi, 2014).

### Seed morphospace extension 2: towards the seed functional spectrum

To gain insights into how key morphological traits relate to seed functions (Saatkamp *et al.*, 2019), we expanded our dataset by including supplementary variables (Table 2). For each family, we collated quantitative continuous data for key seed functions: dispersal (mean dispersal distance), persistence (soil seed bank persistence and recalcitrance probability), and germination (dormancy classes) and climatic variables as important indicators of the plant niche. Although none of these variables contributed to the construction of the morphospace, this approach allows us to examine if they covary with the morphological traits used for its construction (Fig. 2C). Quantitative traits available at the species level were averaged for each family (mean seed dispersal distance, recalcitrance probability and climatic variables). For soil seed bank persistence and dormancy classes, we calculated the proportion of species in each family that have persistence or dormancy. The inclusion of these supplementary variables is not exhaustive, but serves as a proof-of-concept to illustrate how the seed morphospace can be used to retrieve relevant information on seed function and ecological correlates.

The first dimension of this functional extension of the seed morphospace can be interpreted as a ‘dispersal and persistence axis’. This axis reflects variation in fruit type and seed mass, which appears to be strongly associated with mean seed dispersal distance, soil seed bank persistence and seed recalcitrance. As such, this axis quantifies the trade-off between higher resource allocation to regenerative structures and dispersal ability associated with warmer, humid climates on the right (Fig. 2C), and the persistence in the environment associated with cooler and drier seasonal climates on the left (Moles *et al.*, 2005; Chen *et al.*, 2020; Gioria *et al.*, 2020; Huss and Gierlinger, 2021). The second dimension constitutes the ‘germination axis’ reflecting variation in embryo size ratio and the main dormancy classes. The storage of nutrient reserves in other tissue, rather than in the embryo, has been suggested to act on germination timing, where larger embryo size ratios are usually associated with more rapid germination (Verdú, 2006; Vandelook *et al.*, 2012, 2021) or whose germination timing in the environment is under a physiological (PD) or physical dormancy control (PY), here both aligned with higher climate seasonality (top Fig. 2C) as shown in Rosbakh *et al.* (2023). To test the orthogonality of these two functional axes and to better understand the functional significance of given combinations of morphological traits, we should further expand the collation of functional data, such as the mechanical properties of seeds (e.g. endosperm weakening and fruit coat constraints; Steinbrecher and Leubner, 2018) and more detailed information on seed production, dispersal, persistence and germination (Primack, 1987; Baskin and Baskin, 2014; Long *et al.*, 2015; Thomson *et al.*, 2018; Chen *et al.*, 2020; Carta *et al.*, 2022*a*; Pausas and Lamont, 2022).

## CHALLENGES AND PERSPECTIVES OF THE SEED MORPHOSPACE

### Challenges

This first example of an angiosperm-wide seed morphospace is the result of scoring and synthesizing the diversity of seed forms at the family level. The first challenge will be to translate this approach to the species level, which is the desirable level to conduct robust macroevolutionary research studies (Garamszegi, 2014; Sauquet and Magallón, 2018). To facilitate the compilation of high-quality standardized morphological data at the species level, a first prerequisite would be the development of unbiased phylogenetically and geographically wide morphological trait databases (Sauquet and Magallón, 2018; Saatkamp *et al.*, 2019; Visscher *et al.*, 2022), which ideally should include data on fossils to be integrated into the investigation of the temporal evolution of seeds (Friis *et al.*, 2015; Shi *et al.*, 2021; López-Martínez *et al.*, 2023).

The peculiarity of seed phenotyping is that not all seed traits are accessible for conventional methods (e.g. light microscopy). Thus, while manual dissection and evaluation of morphological traits remain relevant (Dayrell *et al.*, 2023), data quantity and quality could be enhanced by the use of modern technologies [X-ray computed tomography, Hu *et al.* (2020); hyperspectral imaging, Feng *et al.* (2019); magnetic resonance imaging, Borisjuk *et al.* (2023)] that allow quantitative and non-invasive assessment of internal seed/fruit characteristics in 2D, 3D and 4D (structure, composition, function).

There are additional challenges for data scoring related to the morphophysiological variation that seeds encounter during their journey from maturation to germination. Before and after dispersal, embryos can grow inside the seeds (e.g. Ranunculaceae; Ali *et al.*, 2007) and seed/fruit morphological and mechanical properties can change as a result of ripening (e.g. seed coat colour in Brassicaceae; Luzuriaga *et al.*, 2006). Thus, the time-since-dispersal when measurements are made should be considered when scoring seed and fruit traits. In addition, sometimes data are gathered from seed lots stored in conservation seed banks, where the seed banking procedures (e.g. external structures lost as part of the cleaning process such as eso- and mesocarp in fleshy fruits) and the physiological state of the seeds (e.g. freshly collected versus dried seeds) might impact on the measurement (e.g. seed mass data from the Seed Information Database; https://ser-sid.org/). In such cases, the storage history of the seeds should also be taken into account.

Finally, how to score, measure and analyse traits that are only present in particular lineages depend on the study question (Vogt, 2017; Gerber, 2019; Budd, 2021). For instance, the identification of homologous traits becomes crucial if our scope is to understand seed evolution in angiosperms.

### Perspectives

The availability of open-access trait databases and of modern analytical tools provides an unprecedented opportunity to push forward our understanding of the integration and evolution of seed traits. In this context, we envisage three main critical areas for future research of the seed morphospace which may flow into the study of angiosperms’ reproductive system as a whole.

### Expanding the set of seed morphological traits

By focusing on a few key morphological traits, we provide a baseline to further expand the seed morphospace and include additional traits towards a more comprehensive morphological and functional characterization of seed variation. For instance, in angiosperms, endosperm formation occurs after double fertilization, which is considered one main factor underpinning their diversification (Endress, 2011). Thus, in addition to the key traits used here, a secondary set of morphological, anatomical and developmental traits (Carvalho *et al.*, 2023) could be considered, for example: integument number (i.e. outer layers of the ovule, developing into the seed coat after fertilization of the ovule; Johri *et al.*, 1992; Linkies *et al.*, 2010), endosperm formation (cellularization patterns; Geeta, 2003; Linkies *et al.*, 2010), seed set (i.e. number of seeds per fruit; Primack, 1987; Bawa *et al.*, 2019) and dispersal structure investment (Thomson *et al.*, 2018). The inclusion of such traits is crucial to better understand whether a given trait can limit or channel evolvability in others.

### Understanding the drivers of variation in seed forms

Morphospaces do not capture the processes that actually generate those forms; that is, they do not measure the fitness of specific forms, but rather their abundance and occupancy across taxonomic groups. Thus, the seed morphospace should be coupled with macroevolutionary comparative approaches (Garamszegi, 2014) to test hypotheses on the drivers of seed form evolution. For instance, understanding such drivers requires quantification of the tempo (evolutionary rates) and mode (evolutionary process) of seed trait (co)evolution. To achieve this goal, we should evaluate how seed traits are phylogenetically structured across angiosperms and which (if any) are the major evolutionary trends by reconstructing the ancestral history of traits. Then, we should assess what constrains seed evolvability, testing the idea that there are close evolutionary relationships among seed components mediated by their morpho-developmental continuity and ultimately shaped by selective pressures (e.g. Chen *et al.*, 2020; Carta *et al.*, 2022*b*). This multivariate approach, coupled with modelling of diversification rates, and ideally with the inclusion of data from fossil species, could refine our understanding of the macroevolution of seeds and the role of functional acquisition of morphological innovation in angiosperm diversification.

### Providing an integrated view of the sexual reproduction biology of angiosperms

The insights from the seed morphospace can feed into a broader trait network, including vegetative and reproductive traits (Wright *et al.*, 2004; Weemstra *et al.*, 2016; Vojtko *et al.*, 2022), offering the opportunity for comparing patterns and related processes with the spectrum of plant form and function (Díaz *et al.*, 2016). In particular, a critical area of future research should focus on specific biological systems or organs (Verdú and Gleiser, 2006; Hoyle *et al.*, 2015). Specifically, it would be highly desirable to assess whether the seed morphospace is orthogonal (or not) to the flower morphospace (Chartier *et al.*, 2014; Roddy *et al.*, 2021), which can point towards possible evo-devo constraints and trade-offs. Such an approach holds the potential to bring plant science focused on sexual reproduction biology (from flowering to regeneration by seeds) to the frontiers of macroevolutionary studies, possibly contributing to elucidating the drivers of angiosperms’ evolutionary success.

## CONCLUSIONS

The seed morphospace is an ideal tool to detect patterns of covariation among dispersal unit traits, providing a powerful approach for comparing the seed morphology of different species and lineages. Although challenges need to be addressed to untap the full potential of this approach, it may shed light on and generate new hypotheses about the potential drivers of seed evolution. Moreover, in light of ongoing rapid global climate change and the role seeds play in many aspects of biology and nature-based solutions (e.g. food security, ecological restoration and reforestation; Mattana *et al.*, 2022), it is particularly timely and necessary to monitor and understand seed trait variability and its links to the environment (Baskin and Baskin, 2022; Armstrong *et al.*, 2023).

## SUPPLEMENTARY DATA

Supplementary data are available at *Annals of Botany* online and consist of the following.

Figure S1. Projection of angiosperm families (dots) onto the plane defined by the first two dimensions of the seed morphospace, separately per each angiosperm group. Figure S2. Disparity indices calculated for major APG groups. Mean pairwise distances (circles) and range (squares). Table S1. Data matrix used to construct the seed morphospace.

mcae099_suppl_Supplementary_Figure_S1

mcae099_suppl_Supplementary_Figure_S2

mcae099_suppl_Supplementary_Table_S1

## Data Availability

All data supporting this study are available at the original published sources listed in this article and in the Supplementary Data.

## References

[CIT0001] Ali N , Probert R, Hay F, Davies H, Stuppy W. 2007. Post-dispersal embryo growth and acquisition of desiccation tolerance in *Anemone nemorosa* L. seeds. Seed Science Research 17: 155–163.

[CIT0002] APGIV. 2016. An update of the Angiosperm Phylogeny Group classification for the orders and families of flowering plants: APG IV. Botanical Journal of the Linnean Society 181: 1–20.

[CIT0003] Armstrong EM , Larson ER, Harper H, et al 2023. One hundred important questions facing plant science: an international perspective. New Phytologist 238: 470–481.

[CIT0004] Arshad W , Sperber K, Steinbrecher T, et al 2019. Dispersal biophysics and adaptive significance of dimorphic diaspores in the annual *Aethionema arabicum* (Brassicaceae). New Phytologist 221: 1434–1446. 30230555 10.1111/nph.15490 PMC6492137

[CIT0005] Baken E , Collyer M, Kaliontzopoulou A, Adams D. 2021. geomorph v4.0 and gmShiny: enhanced analytics and a new graphical interface for a comprehensive morphometric experience. Methods in Ecology and Evolution 12: 2355–2363.

[CIT0096] Barthlott W. 1981. Epidermal and seed surface characters of plants: systematic applicability and some evolutionary aspects. Nordic Journal of Botany 1: 345–355.

[CIT0006] Baskin CC , Baskin JM. 2014. Seeds: ecology, biogeography, and, evolution of dormancy and germination. Amsterdam: Elsevier.

[CIT0007] Baskin CC , Baskin JM. 2019. Martin’s peripheral embryo - unique but not a phylogenetic ‘orphan’ at the base of his family tree: a tribute to the insight of a pioneer seed biologist. Seed Science Research 29: 155–166.

[CIT0008] Baskin CC , Baskin JM. **eds.** 2022. Plant regeneration from seeds: a global warming perspective. Cambridge, MA: Academic Press.

[CIT0009] Bawa KS , Ingty T, Revell LJ, Shivaprakash KN. 2019. Correlated evolution of flower size and seed number in flowering plants (monocotyledons). Annals of Botany 123: 181–190. 30165602 10.1093/aob/mcy154 PMC6344089

[CIT0010] Beldade P , Koops K, Brakefield PM. 2002. Developmental constraints versus flexibility in morphological evolution. Nature 416: 844–847. 11976682 10.1038/416844a

[CIT0011] Benedict JC , Smith SY, Specht CD, et al 2016. Species diversity driven by morphological and ecological disparity: a case study of comparative seed morphology and anatomy across a large monocot order. AoB Plants 8: plw063. 27594701 10.1093/aobpla/plw063 PMC5091906

[CIT0012] Benton MJ , Wilf P, Sauquet H. 2022. The Angiosperm Terrestrial Revolution and the origins of modern biodiversity. New Phytologist 233: 2017–2035. 34699613 10.1111/nph.17822

[CIT0013] Bobrov AVC , Romanov MS. 2019. Morphogenesis of fruits and types of fruit of angiosperms. Botany Letters 166: 366–399.

[CIT0014] Borisjuk L , Horn P, Chapman K, Jakob PM, Gündel A, Rolletschek H. 2023. Seeing plants as never before. New Phytologist 238: 1775–1794. 36895109 10.1111/nph.18871

[CIT0015] Budd GE. 2021. Morphospace. Current Biology 31: R1181–R1185. 34637728 10.1016/j.cub.2021.08.040

[CIT0016] Carta A , Fernández-Pascual E, Gioria M, et al 2022a. Climate shapes the seed germination niche of temperate flowering plants: a meta-analysis of European seed conservation data. Annals of Botany 129: 775–786. 35303062 10.1093/aob/mcac037 PMC9292614

[CIT0017] Carta A , Mattana E, Dickie J, Vandelook F. 2022b. Correlated evolution of seed mass and genome size varies among life forms in flowering plants. Seed Science Research 32: 46–52.

[CIT0018] Carvalho JDTD , Leme EM, Mariath JEDA. 2023. The seed coat in the evolutionary context of Bromelioideae (Bromeliaceae): morphoanatomical diversity and ontogeny in the core and tankless lineages. Botanical Journal of the Linnean Society 202: 23–51.

[CIT0019] Chartier M , Jabbour F, Gerber S, et al 2014. The floral morphospace – a modern comparative approach to study angiosperm evolution. New Phytologist 204: 841–853. 25539005 10.1111/nph.12969 PMC5526441

[CIT0020] Chen S-C , Wu L-M, Wang B, Dickie JB. 2020. Macroevolutionary patterns in seed component mass and different evolutionary trajectories across seed desiccation responses. New Phytologist 228: 770–777. 32463920 10.1111/nph.16706

[CIT0021] Clark JW , Hetherington AJ, Morris JL, et al 2023. Evolution of phenotypic disparity in the plant kingdom. Nature Plants 9: 1618–1626. 37666963 10.1038/s41477-023-01513-x PMC10581900

[CIT0022] Corner EJH. 1976. The seeds of dicotyledons: volume 1. Cambridge: Cambridge University Press.

[CIT0023] Dayrell RLC , Ott T, Horrocks T, Poschlod P. 2023. Automated extraction of seed morphological traits from images. Methods in Ecology and Evolution 14: 1708–1718.

[CIT0024] Díaz S , Kattge J, Cornelissen JHC, et al 2016. The global spectrum of plant form and function. Nature 529: 167–171. 26700811 10.1038/nature16489

[CIT0025] Endress PK. 2011. Evolutionary diversification of the flowers in angiosperms. American Journal of Botany 98: 370–396. 21613132 10.3732/ajb.1000299

[CIT0026] Eriksson O , Friis EM, Löfgren P. 2000. Seed size, fruit size, and dispersal systems in angiosperms from the Early Cretaceous to the Late Tertiary. The American Naturalist 156: 47–58. 10.1086/303367 10824020

[CIT0027] E-Vojtkó A , Junker RR, de Bello F, Götzenberger L. 2022. Floral and reproductive traits are an independent dimension within the plant economic spectrum of temperate central Europe. New Phytologist 236: 1964–1975. 35842785 10.1111/nph.18386

[CIT0028] Feng L , Zhu S, Liu F, He Y, Bao Y, Zhang C. 2019. Hyperspectral imaging for seed quality and safety inspection: a review. Plant Methods 15: 91. 31406499 10.1186/s13007-019-0476-y PMC6686453

[CIT0029] Finch‐Savage WE , Leubner‐Metzger G. 2006. Seed dormancy and the control of germination. New Phytologist 171: 501–523. 16866955 10.1111/j.1469-8137.2006.01787.x

[CIT0030] Floyd SK , Friedman WE. 2000. Evolution of endosperm developmental patterns among basal flowering plants. International Journal of Plant Sciences 161: S57–S81.

[CIT0031] Fogliani B , Gâteblé G, Villegente M, et al 2017. The morphophysiological dormancy in *Amborella trichopoda* seeds is a pleisiomorphic trait in angiosperms. Annals of Botany 119: 581–590. 28087660 10.1093/aob/mcw244 PMC5379585

[CIT0032] Foote M. 1994. Morphological disparity in Ordovician–Devonian crinoids and the early saturation of morphological space. Paleobiology 20: 320–344.

[CIT0033] Forbis TA , Floyd SK, Queiroz AD. 2002. The evolution of embryo size in angiosperms and other seed plants: implications for the evolution of seed dormancy. Evolution 56: 2112–2125. 12487343 10.1111/j.0014-3820.2002.tb00137.x

[CIT0034] Friis EM , Crane PR, Pedersen KR, Stampanoni M, Marone F. 2015. Exceptional preservation of tiny embryos documents seed dormancy in early angiosperms. Nature 528: 551–554. 26675723 10.1038/nature16441

[CIT0035] Garamszegi LZ. 2014. Modern phylogenetic comparative methods and their application in evolutionary biology: concepts and practice. Berlin: Springer.

[CIT0036] Geeta R. 2003. The origin and maintenance of nuclear endosperms: viewing development through a phylogenetic lens. Proceedings Biological Sciences 270: 29–35. 12590768 10.1098/rspb.2002.2206 PMC1691202

[CIT0037] Gerber S. 2019. Use and misuse of discrete character data for morphospace and disparity analyses. Palaeontology 62: 305–319.

[CIT0038] Gioria M , Pyšek P, Baskin CC, Carta A. 2020. Phylogenetic relatedness mediates persistence and density of soil seed banks. Journal of Ecology 108: 2121–2131.

[CIT0039] Grubb PJ. 1977. The maintenance of species‐richness in plant communities: the importance of the regeneration niche. Biological Reviews 52: 107–145.

[CIT0040] Harper JL , Lovell PH, Moore KG. 1970. The shapes and sizes of seeds. Annual Review of Ecology and Systematics 1: 327–356.

[CIT0041] Holloway T , Steinbrecher T, Pérez M, et al 2021. Coleorhiza-enforced seed dormancy: a novel mechanism to control germination in grasses. New Phytologist 229: 2179–2191. 32970853 10.1111/nph.16948

[CIT0042] Hoyle GL , Steadman KJ, Good RB, McIntosh EJ, Galea LM, Nicotra AB. 2015. Seed germination strategies: an evolutionary trajectory independent of vegetative functional traits. Frontiers in Plant Science 6: 731. 26528294 10.3389/fpls.2015.00731 PMC4600905

[CIT0043] Hu W , Zhang C, Jiang Y, et al 2020. Nondestructive 3D image analysis pipeline to extract rice grain traits using x-ray computed tomography. Plant Phenomics 2020: 3414926. 33313550 10.34133/2020/3414926 PMC7706343

[CIT0044] Huss JC , Gierlinger N. 2021. Functional packaging of seeds. New Phytologist 230: 2154–2163. 33629369 10.1111/nph.17299 PMC8252473

[CIT0045] Jardine PE , Palazzesi L, Tellería MC, Barreda VD. 2022. Why does pollen morphology vary? Evolutionary dynamics and morphospace occupation in the largest angiosperm order (Asterales). New Phytologist 234: 1075–1087. 35147224 10.1111/nph.18024

[CIT0046] Johri BM , Ambegaokar KB, Srivastava PS. 1992. Comparative embryology of angiosperms. Berlin: Springer-Verlag.

[CIT0047] Larson JE , Funk JL. 2016. Regeneration: an overlooked aspect of trait‐based plant community assembly models. Journal of Ecology 104: 1284–1298.

[CIT0048] Lê S , Josse J, Husson F. 2008. FactoMineR: an R package for multivariate analysis. Journal of Statistical Software 25: 1–18.

[CIT0049] Linkies A , Graeber K, Knight C, Leubner‐Metzger G. 2010. The evolution of seeds. New Phytologist 186: 817–831. 20406407 10.1111/j.1469-8137.2010.03249.x

[CIT0050] Long RL , Gorecki MJ, Renton M, et al 2015. The ecophysiology of seed persistence: a mechanistic view of the journey to germination or demise. Biological Reviews of the Cambridge Philosophical Society 90: 31–59. 24618017 10.1111/brv.12095

[CIT0051] López-Martínez AM , Schönenberger J, von Balthazar M, et al 2023. Integrating fossil flowers into the angiosperm phylogeny using a total evidence approach. Systematic Biology 72: 837–855. 36995161 10.1093/sysbio/syad017

[CIT0052] Losos JB. 2008. Phylogenetic niche conservatism, phylogenetic signal and the relationship between phylogenetic relatedness and ecological similarity among species. Ecology Letters 11: 995–1003. 18673385 10.1111/j.1461-0248.2008.01229.x

[CIT0053] Lu L , Fritsch PW, Bush CM, Dong LN, Wang H, Li DZ. 2010. Systematic implications of seed coat diversity in Gaultherieae (Ericaceae). Botanical Journal of the Linnean Society 162: 477–495.

[CIT0054] Luzuriaga AL , Escudero A, Pérez-García F. 2006. Environmental maternal effects on seed morphology and germination in *Sinapis arvensis* (Cruciferae). Weed Research 46: 163–174.

[CIT0055] Martin AC. 1946. The comparative internal morphology of seeds. The American Midland Naturalist 36: 513–660.

[CIT0056] Mattana E , Ulian T, Pritchard HW. 2022. Seeds as natural capital. Trends in Plant Science 27: 139–146. 34556418 10.1016/j.tplants.2021.08.008

[CIT0057] Moles AT. 2018. Being John Harper: Using evolutionary ideas to improve understanding of global patterns in plant traits. Journal of Ecology 106: 1–18.

[CIT0058] Moles AT , Ackerly DD, Webb CO, Tweddle JC, Dickie JB, Westoby M. 2005. A brief history of seed size. Science 307: 576–580. 15681384 10.1126/science.1104863

[CIT0059] Nakabayashi K , Leubner-Metzger G. 2021. Seed dormancy and weed emergence: from simulating environmental change to understanding trait plasticity, adaptive evolution, and population fitness. Journal of Experimental Botany 72: 4181–4185. 34048571 10.1093/jxb/erab150 PMC8163051

[CIT0060] Nikolaeva MG. 2004. On criteria to use in studies of seed evolution. Seed Science Research 14: 315–320.

[CIT0061] Oyston JW , Hughes M, Gerber S, Wills MA. 2016. Why should we investigate the morphological disparity of plant clades? Annals of Botany 117: 859–879. 26658292 10.1093/aob/mcv135 PMC4845799

[CIT0062] Paulsen TR , Högstedt G, Thompson K, Vandvik V, Eliassen S, Leishman M. 2014. Conditions favouring hard seededness as a dispersal and predator escape strategy. The Journal of Ecology 102: 1475–1484. 25558091 10.1111/1365-2745.12323 PMC4277852

[CIT0063] Pausas JG , Lamont BB. 2022. Fire‐released seed dormancy – a global synthesis. Biological Reviews 97: 1612–1639. 10.1111/brv.12855 PMC9540907 35384243

[CIT0064] Primack RB. 1987. Relationships among flowers, fruits, and seeds. Annual Review of Ecology and Systematics 18: 409–430.

[CIT0065] Ramírez-Barahona S , Sauquet H, Magallón S. 2020. The delayed and geographically heterogeneous diversification of flowering plant families. Nature Ecology & Evolution 4: 1232–1238. 32632260 10.1038/s41559-020-1241-3

[CIT0066] Raup DM. 1966. Geometric analysis of shell coiling: general problems. Journal of Paleontology 40: 1178–1190.

[CIT0067] Revell LJ. 2012. phytools: an R package for phylogenetic comparative biology (and other things). Methods in Ecology and Evolution 3: 217–223.

[CIT0068] Roddy AB , Martínez‐Perez C, Teixido AL, et al 2021. Towards the flower economics spectrum. New Phytologist 229: 665–672. 32697862 10.1111/nph.16823

[CIT0069] Rosbakh S , Carta A, Fernández‐Pascual E, et al 2023. Global seed dormancy patterns are driven by macroclimate but not fire regime. New Phytologist 240: 555–564. 37537732 10.1111/nph.19173

[CIT0070] Saatkamp A , Cochrane A, Commander L, et al 2019. A research agenda for seed‐trait functional ecology. New Phytologist 221: 1764–1775. 30269352 10.1111/nph.15502

[CIT0071] Sansalone G , Paolo C, Riccardo C, Stephen W, Silvia C, Pasquale R. 2022. Trapped in the morphospace: the relationship between morphological integration and functional performance. Evolution 76: 2020–2031. 35864587 10.1111/evo.14575 PMC9542761

[CIT0072] Sauquet H , Magallón S. 2018. Key questions and challenges in angiosperm macroevolution. New Phytologist 219: 1170–1187. 29577323 10.1111/nph.15104

[CIT0073] Sauquet H , von Balthazar M, Magallón S, et al 2017. The ancestral flower of angiosperms and its early diversification. Nature Communications 8: 1–10. 10.1038/ncomms16047 PMC5543309 28763051

[CIT0074] Senande-Rivera M , Insua-Costa D, Miguez-Macho G. 2022. Spatial and temporal expansion of global wildland fire activity in response to climate change. Nature Communications 13: 1208. 10.1038/s41467-022-28835-2 PMC8904637 35260561

[CIT0075] Shi G , Herrera F, Herendeen PS, Clark EG, Crane PR. 2021. Mesozoic cupules and the origin of the angiosperm second integument. Nature 594: 223–226. 34040260 10.1038/s41586-021-03598-w

[CIT0076] Sims HJ. 2013. Morphological rates of angiosperm seed size evolution. Evolution 67: 1338–1346. 23617912 10.1111/evo.12057

[CIT0077] Soltis D , Soltis P, Endress P, Chase M, Manchester S, Judd W, Majure L, Mavrodiev E. 2018. Phylogeny and evolution of the angiosperms. Chicago: University of Chicago Press.

[CIT0078] Stebbins GL. 1951. Natural selection and the differentiation of angiosperm families. Evolution 5: 299–324.

[CIT0079] Steinbrecher T , Leubner-Metzger G. 2018. Tissue and cellular mechanics of seeds. Current Opinion in Genetics & Development 51: 1–10. 29571069 10.1016/j.gde.2018.03.001

[CIT0080] Takhtajan A. 1985. Comparative anatomy of seeds, Vol. I. Leningrad: Izdat Nauka.

[CIT0081] Takhtajan A. 1991. Evolutionary trends in flowering plants. New York: Columbia University Press.

[CIT0082] Thomson FJ , Letten AD, Tamme R, Edwards W, Moles AT. 2018. Can dispersal investment explain why tall plant species achieve longer dispersal distances than short plant species? New Phytologist 217: 407–415. 28833231 10.1111/nph.14735

[CIT0083] Vandelook F , Janssens SB, Probert RJ. 2012. Relative embryo length as an adaptation to habitat and life cycle in Apiaceae. New Phytologist 195: 479–487. 22621412 10.1111/j.1469-8137.2012.04172.x

[CIT0084] Vandelook F , Newton RJ, Bobon N, Bohley K, Kadereit G. 2021. Evolution and ecology of seed internal morphology in relation to germination characteristics in Amaranthaceae. Annals of Botany 127: 799–811. 33534902 10.1093/aob/mcab012 PMC8103812

[CIT0085] Verdú M. 2006. Tempo, mode and phylogenetic associations of relative embryo size evolution in angiosperms. Journal of Evolutionary Biology 19: 625–634. 16599937 10.1111/j.1420-9101.2005.00998.x

[CIT0086] Verdú M , Gleiser G. 2006. Adaptive evolution of reproductive and vegetative traits driven by breeding systems. New Phytologist 169: 409–417. 16411943 10.1111/j.1469-8137.2005.01586.x

[CIT0087] Visscher AM , Vandelook F, Fernández-Pascual E, et al 2022. Low availability of functional seed trait data from the tropics could negatively affect global macroecological studies, predictive models and plant conservation. Annals of Botany 130: 773–784. 36349952 10.1093/aob/mcac130 PMC9758304

[CIT0088] Vogt L. 2017. Assessing similarity: on homology, characters and the need for a semantic approach to non‐evolutionary comparative homology. Cladistics 33: 513–539. 34724752 10.1111/cla.12179

[CIT0089] Walker M , Perez M, Steinbrecher T, et al 2021. Molecular mechanisms and hormonal regulation underpinning morphological dormancy: a case study using *Apium graveolens* (Apiaceae). The Plant Journal 108: 1020–1036. 34510583 10.1111/tpj.15489

[CIT0090] Watson L , Dallwitz MJ. 1992 **onwards.** The families of flowering plants: descriptions, illustrations, identification, and information retrieval. delta-intkey.com (1 June 2007).

[CIT0091] Weemstra M , Mommer L, Visser EJW, et al 2016. Towards a multidimensional root trait framework: a tree root review. New Phytologist 211: 1159–1169. 27174359 10.1111/nph.14003

[CIT0092] Werker E. 1997. Seed anatomy. Stuttgart: Gebruder Borntraeger Verlagsbuchhandlung.

[CIT0093] Wright IJ , Reich PB, Westoby M, et al 2004. The worldwide leaf economics spectrum. Nature 428: 821–827. 15103368 10.1038/nature02403

[CIT0094] Wu L-M , Chen S-C, Wang B. 2019. An allometry between seed kernel and seed coat shows greater investment in physical defense in small seeds. American Journal of Botany 106: 371–376. 30866038 10.1002/ajb2.1252

[CIT0095] Wyse SV , Dickie JB. 2017. Predicting the global incidence of seed desiccation sensitivity. Journal of Ecology 105: 1082–1093.

