# Supplementary material for: The seed morphospace, a new contribution towards the multidimensional study of angiosperm sexual reproductive biology
Source: Ann Bot. 2024 Jun 22;134(5):701–10. doi: 10.1093/aob/mcae099 (PMC11560371; doi:10.1093/aob/mcae099)
Supplement: mcae099_suppl_Supplementary_Figure_S1 [file mcae099_suppl_supplementary_figure_s1.pdf]

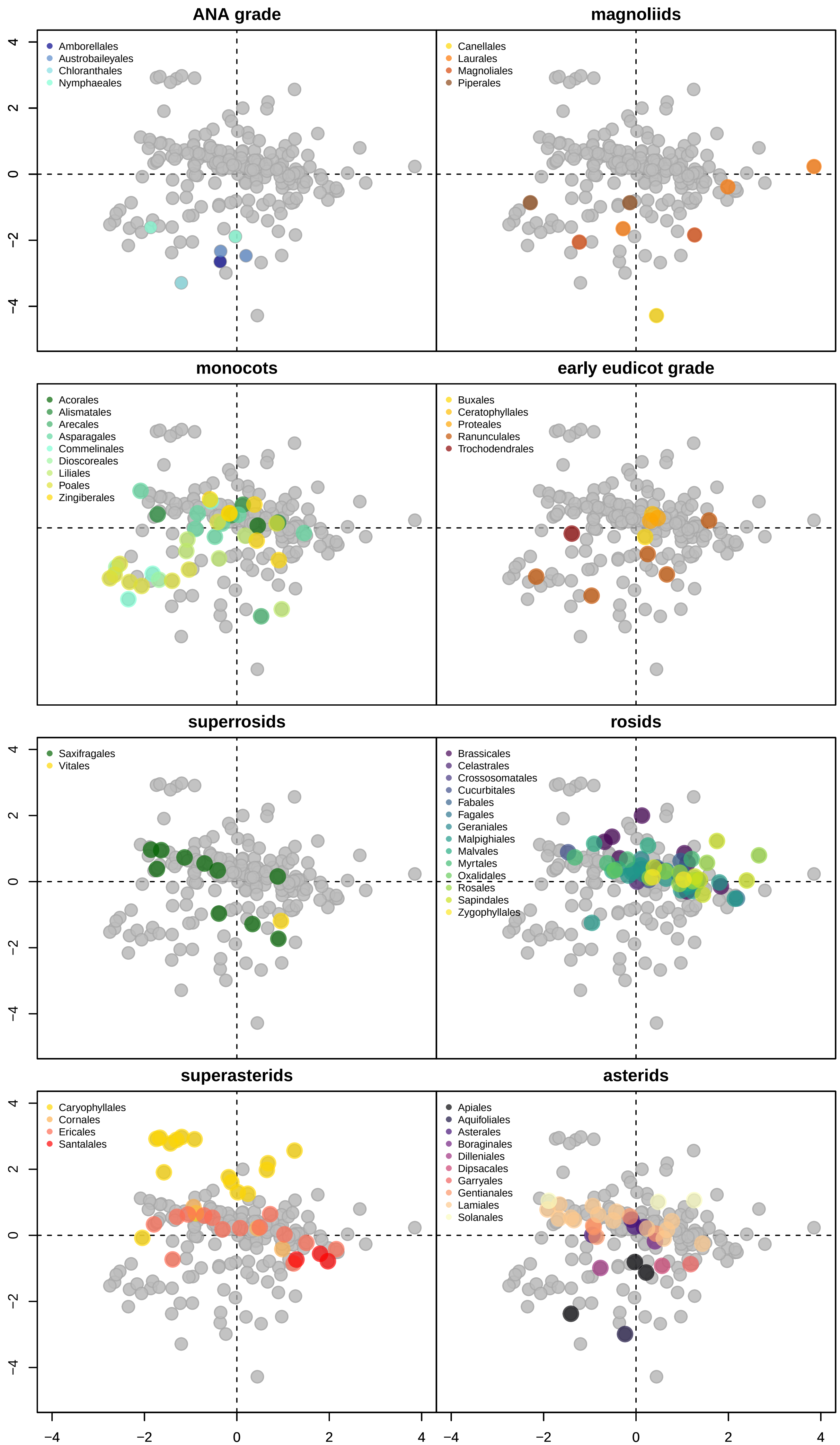
ANA grade
Amborellales
Austrobaileyales
Chloranthales
Nymphaeales
magnoliids
Canellales
Laurales
Magnoliales
Piperales
monocots
Acorales
Alismatales
Arecales
Asparagales
Commelinales
Dioscoreales
Liliales
Poales
Zingiberales
early eudicot grade
Buxales
Ceratophyllales
Proteales
Ranunculales
Trochodendrales
superrosids
Saxifragales
Vitales
rosids
Brassicales
Celastrales
Crossosomatales
Cucurbitales
Fabales
Fagales
Geraniales
Malpighiales
Malvales
Myrtales
Oxalidales
Rosales
Sapindales
Zygophyllales
superasterids
Caryophyllales
Cornales
Ericales
Santalales
asterids
Apiales
Aquifoliales
Asterales
Boraginales
Dilleniales
Dipsacales
Garryales
Gentianales
Lamiales
Solanales
4
2
0
−2
−4
−4
−2
0
2
4
