# Supplementary figures and images for: The seed morphospace, a new contribution towards the multidimensional study of angiosperm sexual reproductive biology
Source: Ann Bot. 2024 Jun 22;134(5):701–10. doi: 10.1093/aob/mcae099 (PMC11560371; doi:10.1093/aob/mcae099)

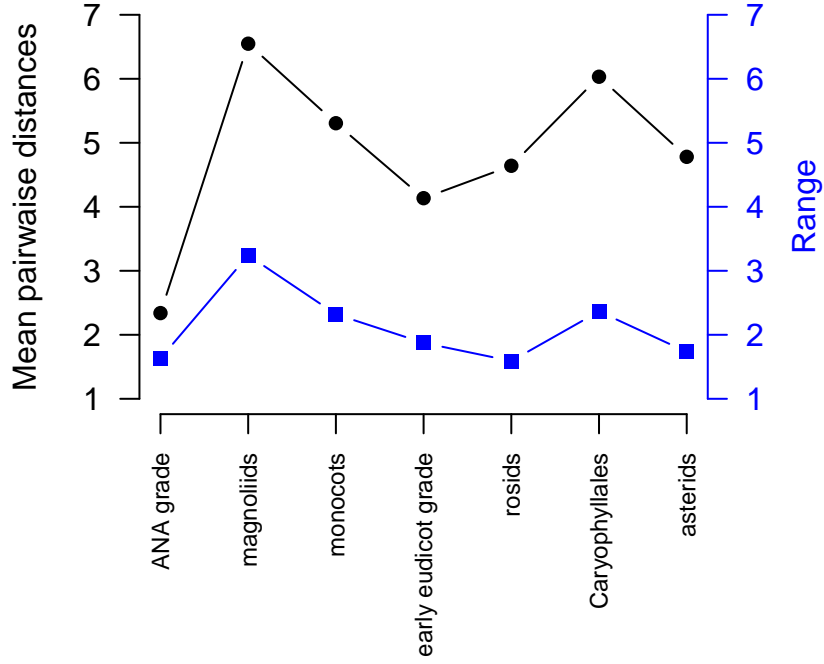

Supplement: mcae099_suppl_Supplementary_Figure_S2 [file mcae099_suppl_supplementary_figure_s2.pdf]
