# Supplementary material for: The seed morphospace, a new contribution towards the multidimensional study of angiosperm sexual reproductive biology
Source: Ann Bot. 2024 Jun 22;134(5):701–10. doi: 10.1093/aob/mcae099 (PMC11560371; doi:10.1093/aob/mcae099)
Supplement: mcae099_suppl_Supplementary_Table_S1 [file mcae099_suppl_supplementary_table_s1.pdf]

**Table S1.** Data matrix used to construct the seed morphospace.

| family | seed mass (log) | embryo size ratio (log) | seed coat ratio (log) | embryo type | seed perispermic | fruit dry dehiscent | fruit dry indehiscent | fruit fleshy |
|---|---|---|---|---|---|---|---|---|
| Acanthaceae | 0.2332 | 0.4050 | NA | Axile | 0 | dehiscent | 0 | fleshy |
| Achatocarpaceae | 0.0492 | 1.5827 | NA | Peripheral | perispermic | 0 | 0 | fleshy |
| Acoraceae | -0.2530 | -0.0048 | NA | Axile | perispermic | 0 | 0 | fleshy |
| Actinidiaceae | -1.0817 | 0.1151 | NA | Axile | 0 | dehiscent | 0 | 0 |
| Aizoaceae | -0.2470 | 1.0389 | NA | Axile | perispermic | dehiscent | indehiscent | fleshy |
| Alismataceae | -0.7401 | 0.7226 | NA | Axile | 0 | 0 | indehiscent | 0 |
| Alstroemeriaceae | 0.3762 | -0.1990 | NA | Axile | 0 | dehiscent | 0 | fleshy |
| Altingiaceae | -0.0946 | 0.3920 | NA | Axile | 0 | dehiscent | 0 | 0 |
| Amaranthaceae | -0.5312 | 1.4729 | 1.0404 | Peripheral | perispermic | dehiscent | indehiscent | 0 |
| Amaryllidaceae | -0.2102 | 0.5512 | NA | Axile | 0 | dehiscent | 0 | fleshy |
| Amborellaceae | -0.1109 | -1.8984 | NA | Basal | 0 | 0 | 0 | fleshy |
| Anacardiaceae | 0.9515 | 0.9421 | 0.3507 | Axile | 0 | 0 | 0 | fleshy |
| Annonaceae | 1.1544 | -1.8984 | -0.6464 | Axile | 0 | 0 | 0 | fleshy |
| Apiaceae | -0.1828 | -1.3152 | NA | Axile | 0 | 0 | indehiscent | 0 |
| Apocynaceae | 0.3343 | 0.2971 | 1.0568 | Axile | 0 | dehiscent | 0 | 0 |
| Aquifoliaceae | 0.2743 | -2.2832 | NA | Basal | 0 | 0 | 0 | fleshy |
| Araceae | 0.5389 | 0.2540 | -1.2559 | Axile | 0 | dehiscent | 0 | fleshy |
| Araliaceae | -0.0929 | -1.8984 | 0.6760 | Basal | 0 | dehiscent | 0 | fleshy |
| Arecaceae | 1.9077 | -1.2158 | 0.8735 | Basal | 0 | 0 | 0 | fleshy |
| Aristolochiaceae | 0.1973 | -1.1690 | NA | Axile | 0 | dehiscent | 0 | fleshy |
| Asparagaceae | 0.1083 | 0.1474 | -1.3493 | Axile | 0 | 0 | 0 | fleshy |
| Asphodelaceae | -0.2812 | 0.1788 | NA | Axile | 0 | dehiscent | 0 | 0 |
| Asteliaceae | -0.6836 | -0.5630 | NA | Axile | 0 | dehiscent | 0 | fleshy |
| Asteraceae | -0.7170 | 0.4557 | 0.4399 | Axile | 0 | 0 | indehiscent | 0 |
| Atherospermataceae | -0.1468 | -2.5322 | NA | Axile | 0 | 0 | indehiscent | 0 |
| Austrobaileyaceae | 1.3461 | -1.7431 | 0.1346 | Basal | perispermic | 0 | 0 | fleshy |
| Balsaminaceae | -0.1400 | 0.4557 | NA | Axile | 0 | dehiscent | 0 | 0 |
| Berberidaceae | 0.3428 | -1.4231 | NA | Axile | 0 | 0 | 0 | fleshy |
| Betulaceae | -0.3420 | 0.4306 | 0.8111 | Axile | 0 | 0 | indehiscent | 0 |
| Bignoniaceae | 0.4729 | 0.4557 | 0.2776 | Axile | 0 | dehiscent | 0 | 0 |
| Bixaceae | 0.7443 | 0.4050 | 0.6452 | Axile | 0 | dehiscent | 0 | 0 |
| Boraginaceae | -0.2042 | 0.3249 | 0.8538 | Axile | 0 | 0 | 0 | fleshy |
| Brassicaceae | -0.7341 | 1.2716 | -0.7963 | Axile | 0 | dehiscent | indehiscent | 0 |
| Bromeliaceae | -0.8240 | 0.0480 | NA | Axile | 0 | dehiscent | 0 | fleshy |
| Burseraceae | 1.3992 | 0.4557 | -0.1532 | Axile | 0 | dehiscent | indehiscent | fleshy |
| Buxaceae | 0.4216 | -0.2418 | NA | Axile | 0 | dehiscent | 0 | fleshy |
| Cabombaceae | -0.1896 | -2.0759 | NA | Basal | perispermic | dehiscent | indehiscent | 0 |
| Cactaceae | -0.4918 | 0.9421 | 0.9975 | Peripheral | perispermic | 0 | 0 | fleshy |
| Calycanthaceae | 1.3821 | 0.4557 | -4.5122 | Axile | 0 | 0 | indehiscent | fleshy |
| Campanulaceae | -1.6090 | -0.4295 | NA | Axile | 0 | dehiscent | 0 | fleshy |
| Canellaceae | 1.5216 | -5.2587 | NA | Axile | 0 | 0 | 0 | fleshy |
| Cannabaceae | 0.7024 | 1.4729 | 0.7325 | Axile | 0 | 0 | indehiscent | fleshy |
| Cannaceae | 1.4000 | 0.1314 | NA | Axile | perispermic | dehiscent | 0 | 0 |
| Capparaceae | 0.8522 | 0.4557 | 0.0489 | Peripheral | 0 | 0 | 0 | fleshy |

| family | seed mass (log) | embryo size ratio (log) | seed coat ratio (log) | embryo type | seed perispermic | fruit dry dehiscent | fruit dry indehiscent | fruit fleshy |
|---|---|---|---|---|---|---|---|---|
| Caprifoliaceae | -0.2376 | -0.9200 | -0.2339 | Axile | 0 | 0 | 0 | fleshy |
| Caricaceae | 0.7554 | 0.3920 | 0.4771 | Axile | 0 | 0 | 0 | fleshy |
| Caryophyllaceae | -1.0491 | 1.3033 | NA | Peripheral | perispermic | dehiscent | indehiscent | 0 |
| Casuarinaceae | -0.2872 | 0.4557 | 0.4858 | Axile | 0 | 0 | indehiscent | 0 |
| Celastraceae | 0.6090 | 0.3522 | 0.9856 | Axile | 0 | dehiscent | 0 | fleshy |
| Centroplacaceae | 1.0200 | -0.4295 | NA | Basal | 0 | dehiscent | 0 | 0 |
| Ceratophyllaceae | -0.2188 | 0.4557 | NA | Axile | 0 | 0 | indehiscent | 0 |
| Cercidiphyllaceae | -0.9044 | 0.4179 | NA | Axile | 0 | dehiscent | 0 | 0 |
| Chloranthaceae | -0.5954 | -3.5385 | NA | Basal | perispermic | 0 | 0 | fleshy |
| Chrysobalanaceae | 2.2775 | 0.4557 | NA | Axile | 0 | 0 | 0 | fleshy |
| Cistaceae | -0.7572 | 1.0695 | NA | Axile | 0 | dehiscent | 0 | 0 |
| Cleomaceae | -0.4216 | 1.2521 | NA | Axile | 0 | dehiscent | 0 | 0 |
| Clethraceae | -1.2888 | 0.2971 | NA | Axile | 0 | 0 | indehiscent | fleshy |
| Colchicaceae | -0.2179 | -0.9582 | NA | Axile | 0 | dehiscent | 0 | 0 |
| Commelinaceae | -0.3283 | -1.0383 | NA | Basal | 0 | dehiscent | 0 | 0 |
| Connaraceae | 1.5250 | 0.4557 | -0.7613 | Axile | 0 | dehiscent | 0 | fleshy |
| Convolvulaceae | 0.3231 | 1.4340 | -1.7806 | Axile | 0 | dehiscent | 0 | fleshy |
| Cornaceae | 0.9677 | 0.3522 | 1.0148 | Axile | 0 | 0 | 0 | fleshy |
| Costaceae | 0.0175 | 0.1314 | NA | Axile | perispermic | dehiscent | indehiscent | 0 |
| Crassulaceae | -1.8487 | 0.4557 | NA | Axile | 0 | dehiscent | 0 | 0 |
| Cucurbitaceae | 0.8333 | 0.4557 | 0.7138 | Axile | 0 | dehiscent | 0 | fleshy |
| Cupressaceae | 0.6308 | 0.4050 | -0.5064 | Axile | 0 | 0 | 0 | 0 |
| Cyperaceae | -0.7820 | -1.0383 | 1.0567 | Basal | 0 | 0 | indehiscent | 0 |
| Cyrillaceae | -0.0493 | 0.3386 | NA | Axile | 0 | dehiscent | 0 | fleshy |
| Daphniphyllaceae | 0.7195 | -1.8183 | -0.2804 | Axile | 0 | 0 | 0 | fleshy |
| Datiscaceae | -1.5414 | 0.4557 | NA | Axile | 0 | dehiscent | 0 | 0 |
| Dilleniaceae | 0.1305 | -1.9840 | -0.0349 | Axile | 0 | dehiscent | indehiscent | 0 |
| Dioscoreaceae | 0.2358 | -1.0803 | 0.3795 | Basal | 0 | dehiscent | 0 | 0 |
| Droseraceae | -1.9677 | -1.0803 | NA | Axile | 0 | dehiscent | 0 | 0 |
| Ebenaceae | 1.3547 | -0.2203 | 0.6154 | Axile | 0 | 0 | 0 | fleshy |
| Elaeagnaceae | 0.5346 | 0.4050 | -0.4733 | Axile | 0 | 0 | indehiscent | 0 |
| Ephedraceae | 0.5870 | 0.4179 | NA | Axile | 0 | 0 | 0 | 0 |
| Ericaceae | -1.0671 | -0.0599 | 1.1202 | Axile | 0 | dehiscent | 0 | 0 |
| Eriocaulaceae | -1.6646 | -1.4809 | NA | Basal | 0 | dehiscent | 0 | 0 |
| Erythroxylaceae | 0.9369 | 0.4557 | 0.4565 | Axile | 0 | 0 | 0 | fleshy |
| Euphorbiaceae | 0.3257 | 0.3920 | -0.1033 | Axile | 0 | dehiscent | indehiscent | fleshy |
| Fabaceae | 0.4207 | 0.8002 | -0.5058 | Axile | 0 | dehiscent | indehiscent | fleshy |
| Fagaceae | 2.1448 | 0.4557 | -0.3075 | Axile | 0 | dehiscent | indehiscent | 0 |
| Fouquieriaceae | 0.1922 | 0.4557 | NA | Axile | 0 | dehiscent | 0 | 0 |
| Frankeniaceae | -1.1938 | 0.4557 | NA | Peripheral | 0 | dehiscent | 0 | 0 |
| Garryaceae | 0.4361 | -0.8120 | NA | Axile | 0 | 0 | indehiscent | fleshy |
| Gelsemiaceae | 0.6390 | 0.2971 | NA | Axile | 0 | dehiscent | 0 | fleshy |
| Gentianaceae | -1.4284 | -0.4551 | NA | Axile | 0 | dehiscent | 0 | fleshy |
| Geraniaceae | -0.2145 | 0.9752 | 0.8703 | Axile | 0 | 0 | indehiscent | 0 |
| Ginkgoaceae | 2.1298 | -0.3089 | NA | Axile | 0 | 0 | 0 | 0 |
| Goodeniaceae | -0.3848 | 0.1788 | 0.0773 | Axile | 0 | 0 | indehiscent | 0 |

| family | seed mass (log) | embryo size ratio (log) | seed coat ratio (log) | embryo type | seed perispermic | fruit dry dehiscent | fruit dry indehiscent | fruit fleshy |
|---|---|---|---|---|---|---|---|---|
| Grossulariaceae | -0.2633 | -1.4231 | NA | Axile | 0 | 0 | 0 | fleshy |
| Gyrostemonaceae | -0.3660 | 1.1358 | NA | Peripheral | 0 | 0 | indehiscent | 0 |
| Haemodoraceae | -0.5441 | -2.1753 | 0.3688 | Basal | 0 | dehiscent | 0 | 0 |
| Haloragaceae | -0.6057 | 0.3789 | NA | Axile | 0 | 0 | indehiscent | fleshy |
| Hamamelidaceae | 0.6912 | 0.3656 | 0.3233 | Axile | 0 | dehiscent | 0 | 0 |
| Heliconiaceae | 1.1603 | 0.1632 | NA | Basal | perispermic | 0 | indehiscent | fleshy |
| Hydrangeaceae | -1.3547 | 0.2393 | NA | Axile | 0 | 0 | indehiscent | fleshy |
| Hydrocharitaceae | -0.4482 | 0.4557 | NA | Axile | 0 | dehiscent | 0 | fleshy |
| Hypericaceae | -1.3693 | 0.4557 | NA | Axile | 0 | 0 | 0 | fleshy |
| Hypoxidaceae | -0.6442 | 0.0307 | NA | Axile | 0 | dehiscent | 0 | fleshy |
| Iridaceae | -0.1468 | -0.4812 | -0.0664 | Axile | 0 | dehiscent | 0 | 0 |
| Juglandaceae | 2.4735 | 0.4557 | 0.9294 | Axile | 0 | 0 | indehiscent | fleshy |
| Juncaceae | -1.6561 | -0.9976 | NA | Basal | 0 | dehiscent | 0 | 0 |
| Koeberliniaceae | 0.0149 | 1.3585 | NA | Axile | 0 | 0 | 0 | fleshy |
| Krameriaceae | 1.0799 | 0.3249 | NA | Axile | 0 | 0 | indehiscent | 0 |
| Lamiaceae | -0.5286 | 0.4050 | 1.1453 | Axile | 0 | dehiscent | 0 | 0 |
| Lardizabalaceae | 0.7914 | -0.8830 | NA | Axile | 0 | dehiscent | 0 | fleshy |
| Lauraceae | 1.7596 | 0.4557 | -0.3101 | Axile | 0 | 0 | 0 | fleshy |
| Liliaceae | -0.1845 | -1.3680 | NA | Axile | 0 | dehiscent | 0 | fleshy |
| Limeaceae | -0.0903 | 1.6882 | NA | Peripheral | perispermic | dehiscent | 0 | 0 |
| Limnanthaceae | 0.0167 | 0.4557 | NA | Axile | 0 | 0 | indehiscent | 0 |
| Linaceae | -0.6220 | 0.3656 | NA | Axile | 0 | dehiscent | indehiscent | fleshy |
| Loasaceae | -0.6502 | 0.6715 | NA | Axile | 0 | dehiscent | 0 | 0 |
| Loganiaceae | 0.1947 | 0.3789 | -0.9969 | Axile | 0 | dehiscent | 0 | 0 |
| Lythraceae | -0.9746 | 0.4557 | 0.3909 | Axile | 0 | dehiscent | 0 | 0 |
| Magnoliaceae | 1.0765 | -1.6721 | 0.8438 | Basal | 0 | dehiscent | indehiscent | 0 |
| Malpighiaceae | 0.7554 | 0.5852 | 0.8916 | Axile | 0 | 0 | indehiscent | fleshy |
| Malvaceae | 0.3394 | 1.0311 | -0.6097 | Axile | 0 | dehiscent | indehiscent | 0 |
| Marantaceae | 0.7452 | 0.7326 | NA | Axile | perispermic | dehiscent | 0 | fleshy |
| Martyniaceae | 0.9797 | 0.4557 | NA | Axile | 0 | 0 | 0 | fleshy |
| Mayacaceae | -1.0183 | -1.6050 | NA | Basal | 0 | dehiscent | 0 | 0 |
| Melanthiaceae | -0.0689 | -1.4231 | NA | Axile | 0 | dehiscent | 0 | 0 |
| Melastomataceae | -1.3188 | 0.4557 | 0.0742 | Axile | 0 | dehiscent | 0 | fleshy |
| Meliaceae | 1.4437 | 0.7024 | -0.7916 | Axile | 0 | 0 | indehiscent | fleshy |
| Menispermaceae | 0.9626 | 1.1430 | NA | Axile | 0 | 0 | 0 | fleshy |
| Menyanthaceae | -0.6999 | 0.0131 | NA | Axile | 0 | dehiscent | indehiscent | fleshy |
| Molluginaceae | -1.4506 | 1.2970 | NA | Peripheral | perispermic | dehiscent | 0 | 0 |
| Montiaceae | -0.8813 | 1.2651 | NA | Peripheral | perispermic | dehiscent | 0 | 0 |
| Moraceae | 0.2161 | 1.1712 | -2.7772 | Axile | 0 | 0 | indehiscent | fleshy |
| Myricaceae | 0.1930 | 0.4557 | NA | Axile | 0 | 0 | indehiscent | fleshy |
| Myrtaceae | -0.2513 | 0.4557 | -2.1461 | Axile | 0 | dehiscent | indehiscent | fleshy |
| Nartheciaceae | -1.7288 | -1.1690 | NA | Basal | 0 | dehiscent | 0 | 0 |
| Nyctaginaceae | 0.1682 | 1.2190 | -2.4638 | Peripheral | perispermic | 0 | indehiscent | 0 |
| Nymphaeaceae | 0.1083 | -1.4809 | NA | Basal | perispermic | 0 | indehiscent | fleshy |
| Ochnaceae | 0.5483 | 0.4557 | -1.4548 | Axile | 0 | 0 | 0 | fleshy |
| Olacaceae | 1.6825 | -0.3089 | NA | Axile | 0 | 0 | indehiscent | fleshy |

| family | seed mass (log) | embryo size ratio (log) | seed coat ratio (log) | embryo type | seed perispermic | fruit dry dehiscent | fruit dry indehiscent | fruit fleshy |
|---|---|---|---|---|---|---|---|---|
| Oleaceae | 0.7554 | 0.0652 | 0.4655 | Axile | 0 | 0 | indehiscent | 0 |
| Onagraceae | -1.1852 | 0.4557 | NA | Axile | 0 | dehiscent | indehiscent | fleshy |
| Opiliaceae | 1.7450 | 0.2393 | NA | Axile | 0 | 0 | 0 | fleshy |
| Orchidaceae | -2.6756 | 0.4557 | NA | Axile | 0 | dehiscent | 0 | 0 |
| Orobanchaceae | -1.1039 | -0.0413 | NA | Axile | 0 | dehiscent | 0 | 0 |
| Oxalidaceae | -0.9935 | 0.2393 | NA | Axile | 0 | dehiscent | 0 | fleshy |
| Paeoniaceae | 1.2297 | -1.4231 | NA | Axile | 0 | dehiscent | 0 | 0 |
| Papaveraceae | -0.8360 | -1.3152 | NA | Basal | 0 | dehiscent | 0 | 0 |
| Passifloraceae | 0.0441 | 0.4179 | 0.7379 | Axile | 0 | dehiscent | 0 | fleshy |
| Paulowniaceae | -1.1270 | 0.3789 | NA | Axile | 0 | dehiscent | indehiscent | 0 |
| Pedaliaceae | -0.2016 | 0.4050 | NA | Axile | 0 | dehiscent | indehiscent | 0 |
| Penthoraceae | -2.2193 | 0.3656 | NA | Axile | 0 | dehiscent | 0 | 0 |
| Phrymaceae | -1.8795 | 0.4179 | NA | Axile | 0 | dehiscent | 0 | 0 |
| Phyllanthaceae | 0.3103 | 0.2540 | 0.1216 | Axile | 0 | 0 | 0 | fleshy |
| Phytolaccaceae | 0.1168 | 1.3033 | NA | Peripheral | perispermic | 0 | 0 | fleshy |
| Picrodendraceae | 0.4327 | 0.4432 | NA | Axile | 0 | 0 | 0 | fleshy |
| Pinaceae | 1.0825 | 0.3111 | 0.6706 | Axile | 0 | 0 | 0 | 0 |
| Pittosporaceae | 0.1288 | -1.7431 | -0.4139 | Axile | 0 | 0 | indehiscent | 0 |
| Plantaginaceae | -0.9892 | 0.3656 | -0.7765 | Axile | 0 | 0 | 0 | fleshy |
| Platanaceae | -0.1888 | 0.1788 | NA | Axile | 0 | 0 | indehiscent | 0 |
| Plocospermataceae | 0.5320 | 0.6183 | NA | Axile | 0 | dehiscent | indehiscent | fleshy |
| Plumbaginaceae | -0.4670 | 0.4557 | NA | Axile | 0 | dehiscent | 0 | 0 |
| Poaceae | -0.5894 | -0.5352 | 0.7047 | Basal | 0 | 0 | indehiscent | 0 |
| Polemoniaceae | -0.7290 | 0.3249 | NA | Axile | 0 | dehiscent | 0 | 0 |
| Polygalaceae | 0.0021 | 0.4557 | NA | Axile | 0 | dehiscent | indehiscent | fleshy |
| Polygonaceae | -0.3369 | 0.6183 | 0.3544 | Peripheral | 0 | 0 | indehiscent | 0 |
| Pontederiaceae | -0.4704 | 0.4557 | NA | Axile | 0 | dehiscent | indehiscent | 0 |
| Portulacaceae | -1.4224 | 1.3403 | -0.1157 | Peripheral | perispermic | dehiscent | 0 | 0 |
| Primulaceae | -0.5980 | 0.2245 | -1.2126 | Axile | 0 | 0 | 0 | fleshy |
| Proteaceae | 0.5448 | 0.5280 | 0.7053 | Axile | 0 | dehiscent | indehiscent | fleshy |
| Ranunculaceae | -0.4208 | -1.8183 | -0.0040 | Basal | 0 | dehiscent | indehiscent | fleshy |
| Resedaceae | -1.0106 | 0.7620 | NA | Axile | 0 | dehiscent | 0 | fleshy |
| Restionaceae | -0.4062 | -1.6721 | 0.9180 | Basal | 0 | dehiscent | indehiscent | 0 |
| Rhamnaceae | 0.1742 | 0.4306 | 0.2060 | Axile | 0 | 0 | indehiscent | fleshy |
| Rhizophoraceae | 0.9737 | 0.4557 | NA | Axile | 0 | dehiscent | 0 | fleshy |
| Rosaceae | 0.1511 | 0.3920 | 0.8652 | Axile | 0 | 0 | indehiscent | fleshy |
| Rubiaceae | -0.0998 | -0.0048 | 0.2997 | Axile | 0 | dehiscent | 0 | 0 |
| Rutaceae | 0.1879 | 0.5739 | 0.4684 | Axile | 0 | dehiscent | indehiscent | fleshy |
| Salicaceae | -0.8009 | 0.3656 | 0.1341 | Axile | 0 | dehiscent | 0 | fleshy |
| Santalaceae | 0.7212 | -0.4812 | 0.2925 | Axile | 0 | 0 | indehiscent | fleshy |
| Sapindaceae | 1.0568 | 1.8022 | -1.0249 | Axile | 0 | dehiscent | indehiscent | fleshy |
| Sapotaceae | 1.9856 | 0.4557 | -0.4110 | Axile | 0 | 0 | 0 | fleshy |
| Sarraceniaceae | -0.5406 | -1.6050 | NA | Axile | 0 | dehiscent | 0 | 0 |
| Saururaceae | -1.2854 | -1.3152 | NA | Basal | perispermic | dehiscent | indehiscent | 0 |
| Saxifragaceae | -1.6972 | -0.3321 | NA | Axile | 0 | dehiscent | 0 | 0 |
| Schisandraceae | 0.2555 | -1.8984 | -0.0516 | Basal | perispermic | 0 | 0 | fleshy |

| family | seed mass (log) | embryo size ratio (log) | seed coat ratio (log) | embryo type | seed perispermic | fruit dry dehiscent | fruit dry indehiscent | fruit fleshy |
|---|---|---|---|---|---|---|---|---|
| Scrophulariaceae | -0.9490 | 0.2393 | 1.1478 | Axile | 0 | dehiscent | 0 | 0 |
| Simaroubaceae | 1.3487 | 0.4179 | 0.4351 | Axile | 0 | 0 | 0 | fleshy |
| Simmondsiaceae | 1.7562 | 0.4557 | NA | Peripheral | 0 | dehiscent | 0 | 0 |
| Smilacaceae | 0.8179 | -1.6050 | -1.8346 | Basal | 0 | 0 | 0 | fleshy |
| Solanaceae | -0.4507 | 1.1358 | -0.2971 | Axile | 0 | dehiscent | indehiscent | fleshy |
| Sphenocleaceae | -2.3144 | 0.4557 | NA | Axile | 0 | dehiscent | 0 | 0 |
| Staphyleaceae | 0.9241 | 0.3789 | NA | Axile | 0 | dehiscent | indehiscent | fleshy |
| Strelitziaceae | 1.3923 | 0.1151 | NA | Axile | perispermic | dehiscent | 0 | 0 |
| Styracaceae | 1.3667 | 0.4306 | 0.9910 | Axile | 0 | dehiscent | 0 | 0 |
| Symplocaceae | 0.9326 | 1.1850 | 0.9651 | Axile | 0 | dehiscent | indehiscent | fleshy |
| Taxaceae | 1.4626 | -0.5916 | NA | Axile | 0 | 0 | 0 | 0 |
| Tetrachondraceae | -2.2151 | 0.0987 | NA | Axile | 0 | dehiscent | 0 | 0 |
| Theaceae | 0.8804 | 0.4179 | -0.3191 | Axile | 0 | 0 | 0 | fleshy |
| Thymelaeaceae | 0.0218 | 0.3920 | -0.5337 | Axile | 0 | 0 | 0 | fleshy |
| Tofieldiaceae | -1.6843 | -0.2862 | NA | Axile | 0 | dehiscent | 0 | 0 |
| Trochodendraceae | -0.8411 | -0.8830 | NA | Axile | 0 | dehiscent | 0 | 0 |
| Tropaeolaceae | 1.1193 | 0.4557 | NA | Axile | 0 | 0 | indehiscent | fleshy |
| Typhaceae | -0.6356 | 0.3386 | NA | Axile | 0 | 0 | indehiscent | fleshy |
| Ulmaceae | 0.2846 | 0.4557 | NA | Axile | 0 | 0 | indehiscent | 0 |
| Urticaceae | -0.8146 | 0.4179 | 1.1635 | Axile | 0 | 0 | indehiscent | fleshy |
| Verbenaceae | -0.3300 | 0.4306 | NA | Axile | 0 | dehiscent | 0 | 0 |
| Violaceae | -0.4721 | 0.2685 | -0.2059 | Axile | 0 | dehiscent | 0 | fleshy |
| Vitaceae | 0.7785 | -0.9582 | 0.1304 | Axile | 0 | 0 | 0 | fleshy |
| Xyridaceae | -1.7733 | -1.6721 | NA | Basal | 0 | dehiscent | 0 | 0 |
| Zamiaceae | 2.9940 | 0.2685 | -2.5596 | Axile | 0 | 0 | 0 | 0 |
| Zingiberaceae | 0.3368 | -0.4044 | 0.6088 | Axile | perispermic | 0 | 0 | fleshy |
| Zygophyllaceae | 0.2726 | 0.2685 | 0.5334 | Axile | 0 | 0 | indehiscent | 0 |
